# Noodles and Newspapers

**Published:** 1891-05-23

**Authors:** 


### The Hospital

A Weekly Journal of

**Science, Medicine, Nursing, and Philanthropy.**

*For Hospitals, Asylums, Infirmaries, Dispensaries, Medical Practitioners, Students, Nurses, and the Charitable Public.*

Vol. X.—No. 243.] [May 23, 1891.

### Noodles and Newspapers.

What is the best method of dealing with stupid people? A really good answer to this question would add almost as much to the happiness of the world as the discovery of a new continent with a fine climate and excellent soil, and without human inhabitants or wild beasts. What a satisfaction it would be, for example, to bring one's clergyman to book after a foolish Sunday morning's sermon! The pleasure of putting him into a moral and material corner, and of keeping him imprisoned there until he should justify all his extraordinary propositions by sound logic would be exceedingly great. Not less would be the mental gratification of pulling up the doctor in the midst of his wise head-shakings and dogmatic asseverations of medical wisdom, and asking him to give a reason for the hope of cure that is in him. The number of people who carry on an extensive daily business without ever stopping to ask themselves how much of rhyme or of reason there may be in their proceedings would not be easily computed.

Several newspaper editors and proprietors have sought our counsel and help in certain difficulties they have with the Post Office authorities. We are loyal subjects of the Queen and supporters of her Government, and our disposition is rather to side with authority than with those who question authority. Still, as free-born Britons we feel that even a government department may be unwise; and if unwisdom can be shown to belong to any particular department, it becomes the highest loyalty to endeavour to root out the foolish policy by all constitutional and lawful means.

The complaint of the newspaper proprietors who have appealed to us is that there is one law for the great newspaper and another for the small. A further complaint is that the officials who administer the unequal law wilfully and without reason intensify its inequalities. The first question one naturally asks is—Can these things be true? And the second is—If they are true, how soon can such irritating stupidities be knocked on the head? Governments must remember that whilst England is pretty evenly divided between Tories and Liberals there is nothing which so soon drives an Englishman into the enemy's camp as being kicked without reason in his own.

We have, it is said, to deal with a foolish law, foolishly administered. We cannot fix the responsibility of the law itself, either upon the present government, or upon any member of it, because the law dates from 1870. But it may be possible to bring home the responsibility of its method of administration to a particular person, and that person is perhaps the solicitor to the department. Now it is not impossible to imagine that a solicitor, even a government solicitor, may be able to appreciate the logic of facts; and it is not impossible to imagine him wise enough to avoid driving intelligent men into his enemy's camp unnecessarily. What are the main facts with which we have to deal in this controversy? Here is a sample of one of them in the concrete form: The spring number of the *Lady's Pictorial* of the present year weighed two pounds and a half, and the newspaper called *Pearson's Weekly* weighed something less than two ounces. For the delivery of the *Lady's Pictorial*, weighing two pounds and a half, the Post Office charged one halfpenny; for the delivery of *Pearson's Weekly*, weighing less than two ounces, the same Post Office charged one penny. The fact put in another way gives this striking result: A ton weight of the spring number of the *Lady's Pictorial* would have gone by post for about thirty-three shillings. To post a ton weight of *Pearson's Weekly* for the same week would have cost more than sixty-five pounds. This, it will be seen, is a sufficiently conspicuous concrete fact. We will not run away from it, but will rather call up our friend the solicitor to the department, and ask him to put on his spectacles and look at it. Perhaps it would be as well to ask Mr. Raikes to look at it too. Here are, side by side, two parcels of equal weight and of similar contents. The proprietor of the one parcel who wishes it to be carried a certain distance by the postal authorities has to pay down beforehand sixty-five golden sovereigns; the proprietor of the other parcel, for carrying it the same distance, is let off with a payment of thirty-three shillings. Both these men are loyal Englishmen, and probably both of them supporters of the present government. What has the right honourable gentleman to say for himself?

Mr. Raikes has his answer ready. "*The Lady's Pictorial*," he says, "is a newspaper, but *Pearson's Weekly* is not." Quite so; but what precisely do you mean by a newspaper, Mr. Postmaster General? "That I cannot exactly tell you," says Mr. Raikes; "but this gentleman," pointing to the solicitor for the department, "will decide the point." Now will it be believed that there is nothing in any of the Post Office regulations to indicate in any precise way what a newspaper is? The decision of this fundamental point is left in the absolute discretion of a mere solicitor to the department; and that although this solicitor's unguided and uncontrolled decision may result in a loss of over sixty pounds a week, or more than three thousand a year to a particular individual, or to hundreds of such individuals. This is in common-sense England, where we pride ourselves on our equal laws.

But surely, says the reader, unfamiliar with all these things, there must be very good reasons somewhere why such laws should be made, and why an irresponsible solicitor to a department should be invested with such extraordinary powers over the pockets and the loyalty of his fellow countrymen. We have no doubt that the solicitor in question, like other gentlemen of his profession, could give fifty reasons, or indeed fifty thousand if he were asked. But one particular fact will show in a moment what such reasons would be worth. The American Post Office authorities carry newspapers in precisely the same way as ours. But instead of pretending to define what a newspaper is they leave the decision of that point to the publishers of the papers, and charge simply according to weight; their rate for all sorts of newspapers and periodicals being an uniform one of about a halfpenny a pound.

The custom and experience of America practically settles the matter for Great Britain in the regions of logic, plain sense, and equal justice. Whatever plausible arguments might be advanced in defence of the absurd and iniquitous system now established in this country might be put forward with equal plausibilty in favour of establishing the same system in America. There is not a single rational ground of any kind for our law, still less for its ridiculous administration. If it be intended to check the spread of objectionable literary matter it is utterly useless. If its object be to limit the publication of advertisements, its purpose is both ridiculous and vicious. What Mr. Raikes should do at once is at least to put a stop to the jurisdiction of his irresponible solicitor. When that is done he will, if he be a wise man, endeavour to secure the amendment of the law. It is plain that there is only onelogical position to take up with regard to the carriage of printed matter by the Post Office. All such matter should be carried at uniform rates, and according to weight or bulk: matter which the Post Office can legitimately object to should not be carried at all. There is nothing which irritates, and properly irritates, the average Englishman so much as manifest injustice at the hands of the law. If that injustice be stupid, purposeless, and generally injurious as well, the Englishman, sooner or later, obeys his natural instincts and sweeps away both the injustice and its perpetrators.

It is hardly our business to teach politics. But it is every man's business to seek a remedy when he suffers, and to sympathise with those who suffer, more particularly when they suffer from preventible causes. We suffer from the present action of the postal authorities by the limitation, the injurious and useless limitation, of our advertisements. But our advertisements, like those of other newspapers, are our life-blood. Any experienced person may see that The Hospital, with its varied and special contents, cannot be produced for the price at which it is sold. Every reader who considers the paper of any value to the medical charities and the public will feel with us that the placing of unnecessary difficulties in the way of its success is an injury, not only to the proprietors of the paper, but to the medical charities which it represents, and to the public, to whom it supplies much-needed information and instruction about those charities. We are prepared, and we trust our readers, too, are prepared, to co-operate with all possible energy in any well-considered scheme for the improvement of the present Post Office regulations, in so far as they affect newspapers.

